# New combinations and synonyms in *Rehderodendron* (Styracaceae)

**DOI:** 10.3897/phytokeys.161.54970

**Published:** 2020-09-25

**Authors:** Wan-Yi Zhao, Peter W. Fritsch, Zhong-Cheng Liu, Qiang Fan, Jian-Hua Jin, Wen-Bo Liao

**Affiliations:** 1 State Key Laboratory of Biocontrol and Guangdong Provincial Key Laboratory of Plant Resources, School of Life Sciences, Sun Yat-sen University, Guangzhou 510275, China Sun Yat-sen University Guangzhou China; 2 Botanical Research Institute of Texas, 1700 University Drive, Fort Worth, Texas 76107, USA Botanical Research Institute of Texas Fort Worth United States of America

**Keywords:** *
Parastyrax
*, *
Pterostyrax
*, *
Rehderodendron
*, Styracaceae, synonyms

## Abstract

We demonstrate with morphological characters that the species *Pterostyrax burmanicus* W.W.Sm. & Farrer and *Parastyrax macrophyllus* C.Y.Wu & K.M.Feng (Styracaceae) are best placed in the genus *Rehderodendron* Hu. *Rehderodendron burmanicum* (W.W.Sm. & Farrer) W.Y.Zhao, P.W.Fritsch & W.B.Liao, **comb. nov.** and *Rehderodendron macrophyllum* (C.Y.Wu & K.M.Feng) W.Y.Zhao, P.W.Fritsch & W.B.Liao, **comb. nov.**, are created. We also provide a lectotype for *R. macrophyllum*. These revisions result in the reduction of *Pterostyrax* Siebold & Zucc. to three species and this genus is no longer considered to be documented from Myanmar. Further, *Parastyrax* W.W.Sm. becomes a monotypic genus comprising only *P. lacei* (W.W.Sm.) W.W.Sm., distributed in Kachin State, northeast Myanmar and Yunnan Province, south-western China.

## Introduction

*Rehderodendron* Hu (Styracaceae) is a genus of 6 to 10 species of trees native to China, Vietnam and Myanmar (Ming 1983; Hwang and Grimes 1996; Fritsch et al. 2001; Fritsch 2004; Zhao et al. 2019). It can be easily distinguished from the other genera of the Styracaceae by its large cylindrical fruit harbouring an endocarp with many irregular rays intruding into the mesocarp (Zhao et al. 2019). Species number and boundaries in the genus are poorly understood. To address this problem, we have been conducting phylogenetic and taxonomic research on *Rehderodendron* since 2017 with the goal of creating a comprehensive taxonomic revision of its species.

In the process of carrying out this research, we have discovered that two species of *Rehderodendron* have been mistakenly assigned to the genera *Pterostyrax* Siebold & Zucc. (Wu and Li 1965) and *Parastyrax* W.W.Sm. (Smith 1920). *Parastyrax*, *Pterostyrax* and *Rehderodendron* share such features as fertile shoots strictly lateral, hypanthium adnate to the ovary wall through the entire length of the ovary wall and upper ovules apotropous and lower ovules epitropous (Fritsch et al. 2001). *Pterostyrax* reportedly differs from the other two genera by, for example, flowers arranged on one side of ultimate inflorescence branches (versus not strictly on one side) and small ribbed or winged fruit with a prominent rostrum and mesocarp absent (versus larger fruit with rostrum not prominent and mesocarp present; Hwang and Grimes 1996; Fritsch et al. 2001). *Parastyrax* reportedly differs from *Rehderodendron* by, for example, petals connate at their bases only (coherent; versus connate distinctly beyond their bases), stamens all subequal to equal in length (versus distinctly unequal), style solid (versus hollow), smaller fruit size, fruit surface with conspicuous lenticels and endocarp without rays intruding into the mesocarp (Hwang and Grimes 1996; Fritsch et al. 2001).

Here we transfer *Pterostyrax burmanicus* W.W.Sm. & Farrer and *Parastyrax macrophyllus* C.Y.Wu & K.M.Feng to the genus *Rehderodendron*, with the creation of two new combinations and a new lectotype. As a result of this study, *Pterostyrax* now contains three species and this genus is no longer considered to be documented from Myanmar. Further, *Parastyrax* becomes a monotypic genus distributed in Kachin State, north-eastern Myanmar and Yunnan Province, south-western China. A detailed table comparing the morphology of the revised *Rehderodendron*, *Pterostyrax* and *Parastyrax* is also provided.

## Materials and methods

This contribution is primarily based on a review of literature and examination of herbarium specimens. Herbarium specimens examined were both the physical specimens at SYS and the digital images of specimens from nine other herbaria (BRIT, E, HITBC, K, KUN, L, P, PE and WUK; herbarium acronyms as in Thiers (2020)). Herbarium numbers are barcode numbers unless otherwise indicated. Where available, physically checked specimens are indicated by “!” after the herbarium acronym and digital images that have been examined are indicated by “image!” appearing after the herbarium accession number or barcode number. Additionally, fieldwork to observe living plants of *Parastyrax macrophyllus* was undertaken in Hekou County by the first author in March 2020. The new combinations proposed herein accord with the rules and recommendations of the International Code of Nomenclature Article 41 and Recommendation 41A (Turland et al. 2018).

## Taxonomic treatment

### Rehderodendron burmanicum

Taxon classificationPlantaeEricalesStyracaceae

(W.W.Sm. & Farrer) W.Y.Zhao, P.W.Fritsch & W.B.Liao comb. nov.

41E8C92C-26AC-57C7-9F74-B2077BD96FA0

urn:lsid:ipni.org:names:77211767-1

Figs 1 , 2A

Pterostyrax burmanicus W.W.Sm. & Farrer, Notes Roy. Bot. Gard. Edinburgh 12: 233. 1920.

#### Type.

Myanmar. Kachin: Myitkyina District, Chipwi Township, Langyang Village, ca. 2100 m elev., 2 Apr 1919, *R. Farrer 803* (holotype: E (E00127295) [image!]).

#### Chinese name.

缅甸木瓜红 meaning “Myanmar rehderodendron”.

#### Distribution, ecology and conservation status.

This species is only known from Chipwi Township, Myitkyina District, east-central Kachin State, Myanmar. It is a deciduous tree growing at ca. 2100 m elevation (7000 feet on label) and is still poorly known. The species flowers from March to April; its fruiting time is not known. Based on the IUCN Red List Criteria (IUCN Standards and Petitions Subcommittee 2019), we suggest a conservation status for this species as DD (Data Deficient).

#### Discussion.

**Figure 1.**
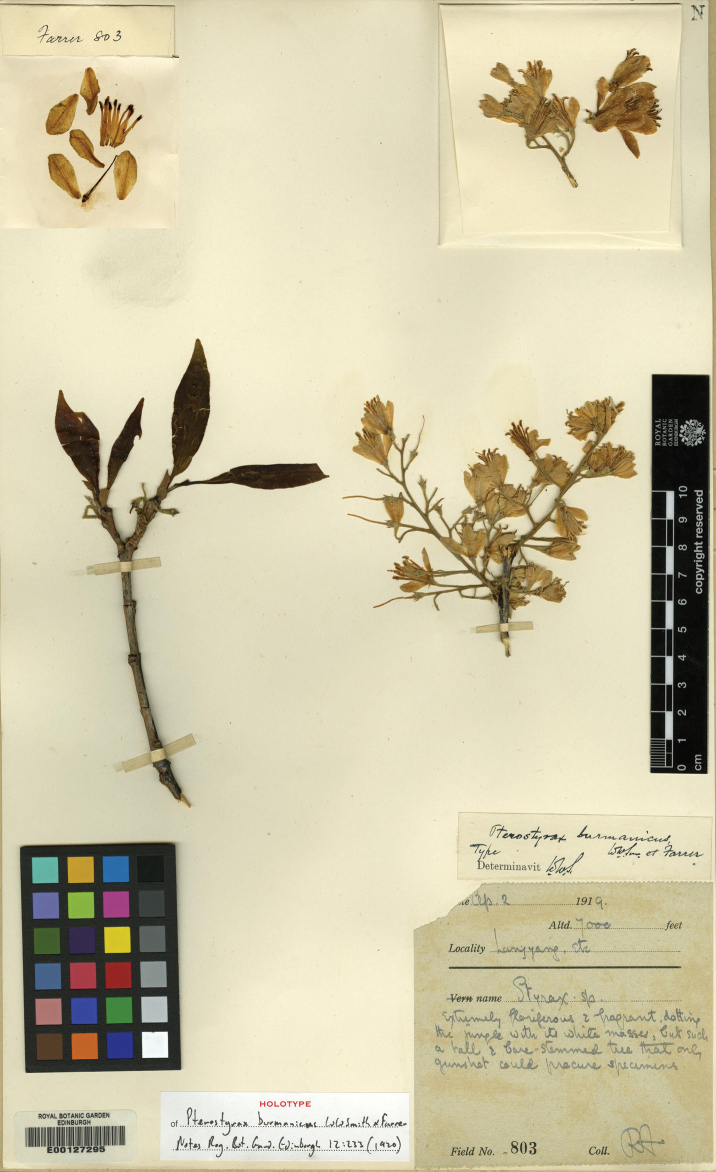
Holotype of *Rehderodendron burmanicum*.

**Figure 2.**
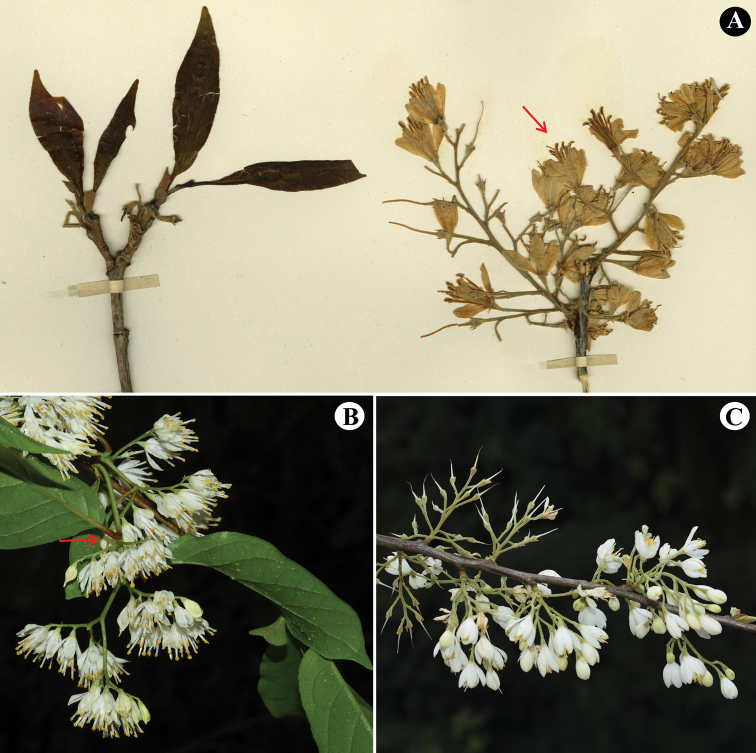
Inflorescences of *Rehderodendron burmanicum*, *Pterostyrax corymbosus* Siebold & Zucc. and *R. microcarpum*. **A** inflorescence of *R. burmanicum*, showing flowering shoots comprising only inflorescences, flowers not strictly arranged on one side of ultimate inflorescence branches and (red arrow) stamens longer than the petals **B** inflorescence of *P. corymbosus*, showing flowering shoots comprising both inflorescences and (red arrow) leaves and flowers arranged on one side of ultimate inflorescence branches **C** inflorescences of *R. microcarpum*, showing flowering shoots comprising only inflorescences, flowers not strictly arranged on one side of ultimate inflorescence branches and stamens that are shorter than the petals. (Photographs: **A** from the holotype of *R. burmanicum***B** by Wan-Yi Zhao from Jinggangshan, Jiangxi, China **C** by Wan-Yi Zhao from Gongshan, Yunnan, China).

*Pterostyrax burmanicus* was described on the basis of a single collection, *R. Farrer 803*, from Langyang, eastern Upper Burma (Smith 1920; Fig. 1). From then until the present work, it had been accepted as a species of *Pterostyrax* Siebold & Zucc. endemic to Myanmar (Wu and Li 1965; Chen and Chen 1996; Kress et al. 2003). The species was not included in the phylogenetic analysis of Fritsch et al. (2001). Although the fruit of *P. burmanicus* is unknown, its vegetative and floral characters all agree with those of *Rehderodendron* (Table 1) and, in combination, match no other genus of the Styracaceae (Hwang and Grimes 1996; Fritsch et al. 2001). Four critical characters differ from those of *Pterostyrax*, but agree with those of *Rehderodendron*: *P. burmanicus* has faintly serrulate leaf blade margins (versus dentate in *Pterostyrax*), fertile shoots comprising only inflorescences (Figs 1, 2A; versus leaves (proximally) and inflorescences (distally); Fig. 2B), flowers that are not strictly arranged on one side of the inflorescence branches (Figs 1, 2A; versus arranged on one side of ultimate inflorescence branches; Fig. 2B) and petals that are distinctly connate beyond their bases (versus connate at their bases only (coherent)). On this basis, we conclude that *P. burmanicus* is best placed in *Rehderodendron* (Table 1).

**Table 1.** Key morphological differences amongst *Rehderodendron*, *Pterostyrax* and *Parastyrax*. N/A = not applicable.

Characters	* Rehderodendron *	* Pterostyrax *	* Parastyrax *
Habit	Evergreen or deciduous	Deciduous	Evergreen
Fertile shoots	Comprising only inflorescences	Comprising both leaves and inflorescences	Comprising only inflorescences
Flower arrangement	Not strictly on one side of the inflorescence branches	On one side of ultimate inflorescence branches	Not strictly on one side of the inflorescence branches
Sepal connation distal to divergence from the hypanthium	Not or only basally connate	Not or only basally connate	Completely connate
Calyx apical margin	Toothed	Toothed	Truncate
Connation of the petals	Distinctly connate beyond their bases	Connate at their bases only	Distinctly connate beyond their bases
Androecium adnation to corolla	Adnate	Adnate	Not adnate
Filament connation	At base or to upper middle	To middle	Nearly completely
Ovary	Incompletely septate	Incompletely septate	Completely septate
Style	Hollow	Hollow	Solid
Stigma shape	Truncate or lobed	Truncate or lobed	Capitate
Fruit length	4–12 cm	1.2–2.5 cm	2.2–3.5 cm
Fruit ribs/wings	5- to 12-ribbed	5- to 10-ribbed or winged	Without ribs
Fruit rostrum	Not prominent or short	Prominent	Not prominent
Fruit exocarp	Without lenticels	Without lenticels	With conspicuous lenticels
Fruit mesocarp presence	Present	Absent	Present
Fruit mesocarp texture	Fleshy/mealy	N/A	Fleshy
Fruit endocarp	With rays intruding into the mesocarp	Without rays intruding into the mesocarp	Without rays intruding into the mesocarp

*Rehderodendron burmanicum* is very similar to *R. microcarpum* K.M.Feng ex T.L.Ming, a species of far north-western Yunnan Province, China and northern Kachin State, Myanmar (Ming 1983; Zhao et al. 2020). Both species are deciduous trees with a paniculate and densely stellate-pubescent inflorescence, filaments connate well below the middle and a pubescent style. The young leaves of *R. burmanicum* have sparsely-scattered stellate pubescence and the stamens are longer than the petals (Figs 1, 2A), whereas the young leaves of *R. microcarpum* are densely covered with stellate pubescence and the stamens are shorter than the petals (Fig. 2C). However, the only known locality of *R. burmanicum* is in Chipwi Township, east-central Kachin State, Myanmar, which is well outside of the known geographic range of *R. microcarpum*. Properly assessing the species boundaries or whether such exist between these two species must await more specimen collections and the generation of morphological and molecular data at the population level.

“*Parastyrax burmanicus* W.W.Sm.” is a name listed in Kress et al. (2003). We could not find any original published literature for this species or digitised specimens identified as such and conclude that it is a nomen nudum, likely resulting from a publication error meant as *Pterostyrax burmanicus*.

### Rehderodendron macrophyllum

Taxon classificationPlantaeEricalesStyracaceae

(C.Y.Wu & K.M.Feng) W.Y.Zhao, P.W.Fritsch & W.B.Liao comb. nov.

2BAEF0D3-D4D3-5912-B118-8794B0FE9818

urn:lsid:ipni.org:names:77211768-1

Figs 3A , 4A–D

Parastyrax macrophyllus C.Y.Wu & K.M.Feng, Yunnan Trop. Subtrop. Fl. Res. Rep. 1: 28, fig. 7. 1965.

#### Type.

China. Yunnan: Hekou County, Binglangzhai, ca. 150 m elev., damp valley, 1 Dec 1959, *Yu Ping-Hua s.n.* (lectotype, designated here: KUN (acc. # KUN0026237) [image!]; isolectotypes: KUN (acc. # KUN0026236) [image!], acc. # KUN0026238 [image!]).

#### Chinese name.

大叶木瓜红 meaning “large-leaved rehderodendron”

**Figure 3.**
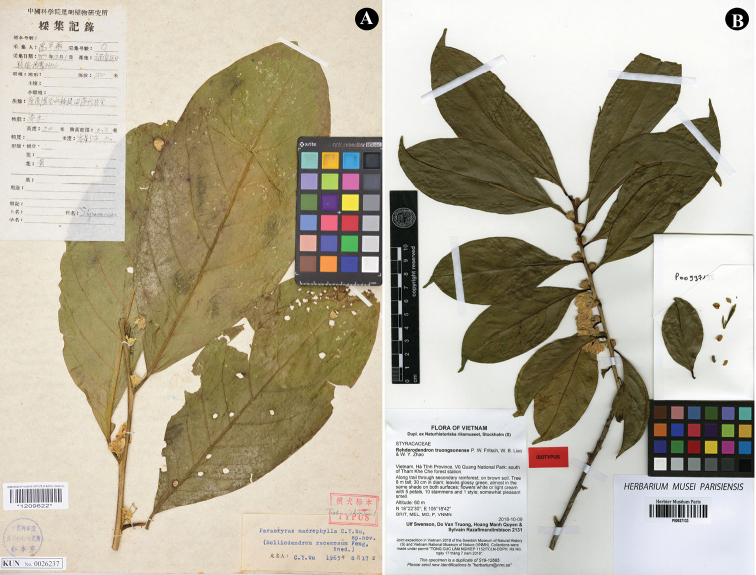
**A** lectotype of *Rehderodendron macrophyllum* (Yu Ping-Hua s.n. [KUN (acc. # KUN0026237)] **B** isotype of *R. truongsonense* (*Ulf Swenson, Do Van Truong, Hoang Manh Quyen & Sylvain Razaflimandimbison 2131* [P00937133]).

#### Distribution, habitat and conservation status.

*Rehderodendron macrophyllum* is known only from Hekou County, Yunnan Province, where three localities of the species (Binglangzhai, Tianweizhai and Shaba) have been documented since 1953. It was recorded as growing in evergreen broad-leaved forests along damp valleys and hillsides at 120–250 m elevation. The original habitat of *R. macrophyllum* is severely disturbed by forestry activities. *Rehderodendron macrophyllum* (as *Parastyrax macrophyllus*) was listed as Critically Endangered by the Chinese government in 2013 (Ministry of Environmental Protection and Chinese Academy of Sciences 2013; Qin et al. 2017). In 2020, we investigated its population, finding only three trees on the hill around Binglangzhai Reservoir, now a protected area. *Rehderodendron macrophyllum* is still seriously threatened and research on the conservation biology of this species is urgently needed. The species flowers from November through January; the mature fruiting time is not known.

#### Discussion.

**Figure 4.**
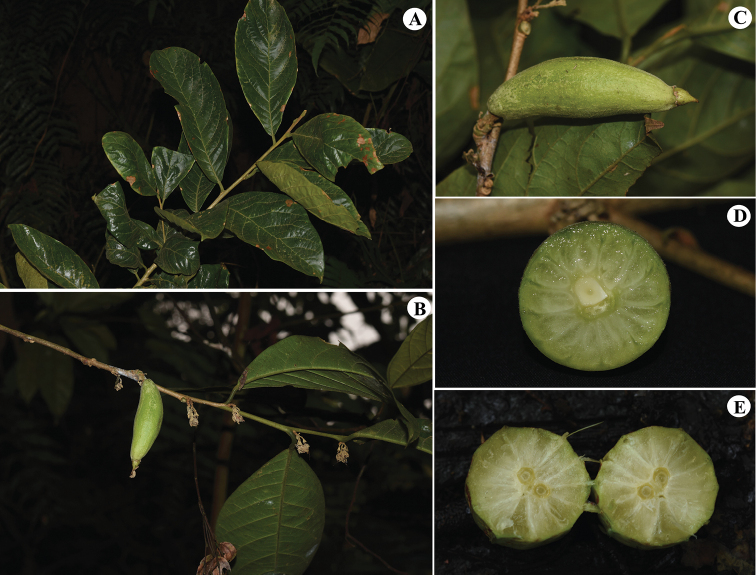
Morphology of *Rehderodendron macrophyllum* and *R. macrocarpum* Hu. **A–D***Rehderodendron macrophyllum***A** branchlet and leaf **B** fruiting branch **C** young fruit **D** transverse section of fruit showing endocarp with many rays intruding into the mesocarp **E** fruit transverse section of *R. macrocarpum*. (Photographs: **A–D** by Wan-Yi Zhao from Hekou, Yunnan, China **E** by Wan-Yi Zhao rom Wawushan, Sichuan, China).

From the time of its original description until the present work, *Parastyrax macrophyllus* had been consistently placed in *Parastyrax*, including in *Flora Yunnanica*, *Flora Reipublicae Popularis Sinicae* and *Flora of China* (Ming 1983; Hwang 1987; Hwang and Grimes 1996). In the original description, Wu and Feng (in Wu and Li 1965) indicated that its inflorescence is very short and its fruit is drupaceous with a fleshy “exocarp” (likely the mesocarp instead), which would be consistent with the characters of *Parastyrax*. We re-examined the original material, including the paratype collection *Cai Ke-Hua 533* (KUN [acc. # KUN0026239!]), which possesses the only fruit gathered of the material cited in the protologue (Wu and Li 1965). We found that the fruit of this collection is clearly immature, so its internal structure, which would otherwise help to confirm the placement of the species to genus, could not be determined. In March 2020, we conducted fieldwork in Binglangzhai, the type locality of *P. macrophyllus*. There, we discovered plants that possess the same vegetative features as those in the type material of the species, yet with fruit in which the endocarp has many irregular rays intruding into the mesocarp, as in *Rehderodendron* (Figs 4D, E) and unlike in *P. lacei* (W.W.Sm.) W.W.Sm., the type species of *Parastyrax*. On this basis, we conclude that *P. macrophyllus* is best placed in *Rehderodendron*. *Parastyrax* must be redefined on characters that exclude those of *R. macrophyllum*; we include diagnostic characters for this purpose in Table 1, based on several previous studies (Smith 1920; Dickison 1993; Hwang and Grimes 1996; Fritsch et al. 2001).

*Rehderodendron macrophyllum* is most similar to *R. truongsonense* P.W.Fritsch, W.B.Liao & W.Y.Zhao (Fig. 3B), a species distributed in northern and central Vietnam (Zhao et al. 2019). These two species share such features as evergreen tree; inflorescences axillary, congested and densely stellate-pubescent; bracteoles foliaceous, elliptic or obovate; filaments connate at base; and style pubescent. However, *R. macrophyllum* can be easily distinguished from *R. truongsonense* by larger leaf blades 12–27 × 6–12(–15) cm (vs. 6–13 × 4–7 cm), with a greater number of lateral veins on each side (8–14 vs. 4–7).

In the protologue, *Yu Ping-hua s.n.*, a flower gathering deposited in KUN, was indicated as the type. Of the three sheets comprising this collection, KUN0026238 and KUN0026237 were stamped as “TYPUS” and KUN0026236 as “ISOTYPUS.” A short annotation is present on KUN0026237, so we have chosen this sheet (Fig. 3A) as the lectotype.

#### Additional specimens examined.

**China. Yunnan**: Hekou County. Binglangzhai, ca. 260 m elev., 12 Dec 1953, *Mao Pin-Yi 3299* (KUN (acc. # KUN0026241) [image!]; PE00857253 [image!]; PE00857254 [image!]; IBSC0453792 [image!]; WUK0205521 [image!]); same locality, ca. 160 m elev., 15 Dec 1953, *Liu Wei-Xin 752* (KUN (acc. # KUN0025120) [image!]; PE00857255 [image!]; HITBC033059 [image!]; LBG00102925); same locality, ca. 120–140 m elev., 2 Nov 1954, *Mao Pin-Yi 5240* (KUN (acc. # KUN0025114) [image!]; PE00857251 [image!]; PE00857250 [image!]; WUK0208259 [image!]); same locality, ca. 120–180 m elev., 8 Nov 1954, *Mao Pin-Yi 5370* (KUN (acc. # KUN0025117) [image!]; PE00857256 [image!]; PE00857257 [image!]; WUK0206253 [image!]); Tianweizhai, ca. 150 m elev., 24 Apr 1953, *Cai Ke-Hua 533* (KUN (acc. # KUN0026239) [image!]; PE00857252 [image!]); Shaba, 17 Dec 1973, *Yang Zeng-Hong 7567* (HITBC033057 [image!]; HITBC033058 [image!]); 25 Nov 1958, *Ding Zhi-Zun & Wang Jia-Xi 1203* (NAS00018374 [image!]); 31 Mar 2020, *Zhao Wan-Yi & Ye Fan ZWY-1563* (SYS!); ditto, *ZWY-1564* (SYS!); ditto, *ZWY-1565* (SYS!).

## Supplementary Material

XML Treatment for Rehderodendron burmanicum

XML Treatment for Rehderodendron macrophyllum
